# Health care workers experiences in emergency obstetric care following implementation of an in-service training program: case of 2 Referral Hospitals in Botswana

**DOI:** 10.4314/ahs.v21i1.9S

**Published:** 2021-05

**Authors:** Ludo Nkhwalume, Yohana Mashalla

**Affiliations:** 1 Institute of Health Sciences, Molepolole, Botswana; 2 Faculty of Medicine, University of Botswana, Gaborone, Botswana

**Keywords:** Maternal mortality, health care workers, EMOC, in-service training

## Abstract

**Background:**

Maternal mortality rate remains a challenge in many developing countries.

**Objectives:**

This study explored experiences of Health Care Workers on Emergency Obstetrics Care (EMOC) in-service training and its effect on maternal mortality.

**Methods:**

Descriptive qualitative study design was conducted using in-depth interviews and focus group discussions. Participants were EMOC trained midwives and doctors purposively selected from the 2 referral hospitals in the country. Data were transcribed verbatim, coded, and analysed using Grounded Theory approach.

**Results:**

Four themes emerged including training, EMOC implementation, maternal death factors and EMOC prioritisation. The duration of training was viewed inadequate but responsiveness to and confidence in managing obstetric emergencies improved post EMOC training. Staff shortage, HCWs non-adherence and negative attitude to EMOC guidelines; delays in instituting interventions, inadequate community involvement, minimal or no health talk to women and their partners and communities on sexual reproductive matters and non-prioritisation of EMOC by authorities were concerns raised.

**Conclusion:**

Strengthening health education at health facility levels, stakeholders' involvement; and prioritising EMOC in-service training are necessary in reducing the national maternal mortality.

## Background

According to the World Health Organisation, global maternal mortality is unacceptably high. In 2017, about 295 000 women died during and following pregnancy and childbirth with majority of the deaths (94%) occurring in low and middle income countries. Most of the deaths could be prevented. Sub-Saharan Africa alone accounted for roughly two-thirds (196 000) of maternal deaths^1^. The risk of maternal mortality is highest for adolescent girls under 15 years old and complications in pregnancy and childbirth are higher among adolescent girls aged 10–19 compared to women aged 20–24^2^,^3^. The major complications that account for nearly 75% of all maternal deaths include severe bleeding, infections, high blood pressure and unsafe abortion^4^. Other complications may exist before pregnancy e.g. diabetes, malaria and cardiovascular diseases but are worsened during pregnancy, especially if not managed as part of the woman's care. The United Nations Sustainable Development Goals (SDGs) has led countries to unite behind a new target to accelerate the decline of maternal mortality by 2030. SDG 3 includes an ambitious target: reducing the global maternal mortality rate (MMR) to less than 70 per 100 000 births, with no country having a maternal mortality rate of more than twice the global average^5^.

In Botswana about 94% of pregnant women attend antenatal care services, 99.8% of the deliveries occur in health facilities and postnatal attendance stands at 85.2%. Yet MMR in the country remains high at 133.7/100,000 6,7. Reports on MMR in Botswana indicate that in 2010, 66% and 16% of the maternal deaths occurred in the two referral hospitals (Princess Marina and Nyangabwe) and district hospitals respectively. Of the reported 80 maternal deaths, 32% died in antenatal period, 11% intra- partum (5 during delivery and 4 during caesarean section), and 52% in postpartum^8^. The most common causes of maternal deaths are haemorrhage, unsafe abortions, eclampsia, sepsis and obstructed labour in that order. Delays in making decisions/referrals and sub-standard care are contributory factors to maternal deaths in Botswana^9^,^10^.

Improving knowledge and skills through training of medical and healthcare staff and maintaining standards of care for emergency obstetric care have been reported to reduce maternal mortality^11^,^12^. Alvarez et al.^13^ and Karlsen, et al.^14^ have reported a correlation between MMR and educational factors and prenatal care coverage. Therefore, intensifying maternal deaths audit has been recommended as an intervention for identifying possible causes of maternal mortality because through maternal death audits improper care are identified leading to timely instituting measures for avoidable maternal deaths^11^.

In 2011, the Botswana Ministry of Health (MOH) intensified Emergency Obstetric Care in-service training and structured maternal death audits as intervention strategies. The training program focused on critical areas including management of obstetric haemorrhage; abortion and anaemia; prolonged obstructed labour and premature labour; hypertensive disorders in pregnancy; puerperal infections and neonatal complications (MOH, Botswana, 2010). Since then the impact of the EMOC training has not been evaluated. The purpose of this study was to explore healthcare workers experiences following emergency obstetric care in-service training & implementation and to identify other contributory factors to maternal mortality in the two referral hospitals

## Methods

### Study sites

The study was conducted in two referral hospitals; Princes Marina Hospital (PMH) in the capital city Gaborone and Nyangabgwe Referral Hospital (NRH). The PMH and NRH are located in the southern and northern part of Botswana respectively. The sites were selected because they admit patients for normal labour and delivery and those requiring specialized care referred from lower level health facilities, have high number of deliveries, live births and maternal deaths than the lower level facilities and are mostly used for EMOC training. This therefore provided an ideal environment for assessing other contributory factors to maternal deaths and obtaining first-hand information on health care workers' experiences.

### Study design

Descriptive qualitative approach was appropriate for this study as it provided the researchers with a comprehensive summary to a phenomenon under study and facilitated generation of findings close to raw data than other qualitative studies^15^.

### Study population

The study population were both male and female adult health care workers (midwives and doctors) trained on EMOC and must have had practiced in obstetrics and gynaecology departments for at least 6 months after training. A list of HCWs trained on EMOC was obtained from the hospitals from which potential participants who met the criteria were purposively selected. These were only five doctors and 32 midwives trained on EMOC in the two referral hospitals

### Data collection

In-depth interviews and focus group discussions (FDGs) were used to collect data. Unstructured qualitative interviews were conducted by the first author. The interview guides were in English, translated by an expert in both languages into Setswana and then back translation to English. The interviews were not audiotaped because of participant's fear that their voices could be identified. Each participant was interviewed individually in a private room by the Primary Investigator. All participants were comfortable being interviewed in English language however they were free to express themselves in the local language. Each interview lasted for 45 to 75 minutes.

Participants for Focus Group Discussions were purposively selected in 3 groups of 6 participants each. FGDs were conducted in private seminar rooms and lasted from 60 to 90 minutes. The Primary Investigator with the use of an interview guide led the guided discussions and sought for clarity as necessary. A trained research assistant took notes and also operated the tape recorder. FGDs were used in order to get first-hand information on perspectives, attitudes and opinions and as complimentary to in-depth interviews on the topic of common interest to participants.

### Consistency and trustworthiness of data

In order to ensure consistency, transparent and clear description of the research process from initial outline, through the development of the methods and reporting of findings were observed. In addition, a research diary documenting challenges and issues assisted the researchers in maintaining cohesion between the study's aim, design and methods. Trustworthiness of the findings was assured through a descriptive qualitative approach which ensured sufficient depth and relevance of data collection and analysis; rich and thick verbatim descriptions of participant's accounts to support the findings were collected and recorded and the first author engaged with peer researchers who had qualitative research expertise during debriefing in order to reduce bias. The investigators regularly, compared notes, evaluated the coding and any differences in the meaning and interpretation of responses were discussed until consensus was reached.

### Ethical consideration

A consent form was designed, written in both the local language (Setswana) and English to allow for free choice for participants. The methods and purposes were explained to participants and then consent forms were given to participants to carefully read before consenting to participate in the study. Participants were informed that participation was voluntary, they were free to withdraw from the study at any time and confidentiality of information would strictly be observed. After careful reading of the consent form, those willing to participate signed the consent. The study received ethical clearance and permission from the Botswana Ministry of Health (Ref: HPD-ME 13/18/1 VII 263).

### Data analysis

Data for both in-depth interviews and FGDs were transcribed verbatim and coded. The Primary investigator listened to the tape recorder several times in order to be familiar with the data. Notes taken and headings were created in the text after which the researcher transcribed the notes and headings onto a coding sheet. The next step involved grouping the data and reducing the number of categories by combining similar headings into broader categories. Data analysis begun from the time of data collection as the researchers interpreted and gave meaning continuously. The emerging themes were identified in line with the objectives of the study and the guiding questions.

## Results

None of the doctors were able to participate in focus group discussions in the 2 hospitals because of work constraints and other assignments. The data generated four main themes: training, EMOC implementation and maternal death factors and government priority setting. The themes are summarized in Table 1.

**Table 1:** Themes, meaning and evidence on EMOC training generated from HCWs in two referral hospitals in Botswana referral hospitals

Themes	Meaning	Evidence
Training	Inadequate	Two weeks is inadequate for this programme
	Improved services delivery	Those trained pick emergencies easily
EMOC implementation	Resistance	Some particularly doctors are reluctant to change
	Low level material	The programme was too low to some
	Inadequate resources	There is shortage of trained staff There is shortage of supplies
Maternal death factors	Patient related factors	Women need to plan for pregnancy to reduce abortions Late presentation to facilities Late registration at ANC
	Facility related factors	Delayed referral decisions at lower level facilities Failure to adhere to protocol by some staff Poor management of patients at lower level facilities
Priority setting	Poor EMOC coordination	Ministry of Health should prioritise EMOC training
	Policy reviews are needed	They need to review old policies in line with current practices

### EMOC training of healthcare workers

Although training was reported to have been intensified, only five doctors and 32 midwives have been trained on EMOC in the two referral hospitals and only one had been trained in 2010 i.e. before training was intensified. Participants indicated that the two weeks duration of training was inadequate for both theory and clinical practice and suggested to be increased from 2 weeks to 1 month. These sentiments were captured as follows:

“*It is a good programme, topics are very relevant and so are practical sessions. It was good to start practising in the skills laboratory using models then going to the clinical area. But, time is the problem, 2 weeks is inadequate for this type of training, if 2 more weeks could be added it can be very beneficial*”.(FGD1)

### Is there a difference in service delivery following EMOC in-service training?

Participants' views were that there is a difference in service delivery as those trained practice according to EMOC protocol and were more responsive to emergencies, have confident in managing post-partum haemorrhage and eclampsia. This was espoused in the following quotes:

HCWM018: “*There is a big difference because if a patient presents with a complication, I act immediately before the doctor arrives. E.g. management of severe PET, initially I couldn't diagnose it, I did not consider severe headache, and epigastric pain; now I know they are indications for sever PET. I know that for a patient with severe PET you catheterize before giving magnesium sulphate to monitor urine output. It is important to know that the patient renal function is ok before administering it, this helps to determine the effect of magnesium on the renal function. PPH was a big problem before EMOC now things have changed; the first thing is to call for help, catheterize the patient, put up iv fluids and give oxytocin per drip and assess to determine where bleeding is from*”

FGD1 “*Majority of those trained have a sense of urgency, they pick emergencies easily and act accordingly. They always follow EMOC guidelines. On the other hand there are some of untrained who are exceptionally good yet they learnt from us who have been trained*”.

Although there is indication of some improvement, findings also indicate that some of the HCWs did not want to follow the guideline.

HCWM001: “*Some are doing their best in what they have been taught. Some particularly doctors are reluctant to change, e.g. vaginal packing during PPH is not supposed to be done but they still do, they refuse to manage patients as per EMOC guidelines*” “*I think it depends more on experience. For some it was useless as it was too low for them, for others it made them follow guidelines blindly and this causes friction. This is so because EMOC should not replace one's clinical judgement*” said HCWD004

### Challenges affecting EMOC Implementation

Both doctors and midwives indicated that there were challenges affecting implementation of EMOC including shortage of staff, delays in referring patients by lower facilities, non-attendance of antenatal care services, refusal to follow EMOC guidelines by some health care workers, delays in instituting interventions, unavailability of some drugs and supplies, negative attitude of some health care workers. These concerns were expressed in the following quotes:

HCW015 said: “*Yes, shortage of EMOC trained manpower because we end up not pulling together. Some accept the guidance from those trained, others are resistant. Shortage of equipment is another challenge e.g. Magnesium sulphate, oxytocin especially when we are to give the maintenance dose. We end up using medications in the “crush cards” emergency trolley*”

“*But generally it is not that bad because most of us who are trained see the importance of EMOC and we still continue doing what we are supposed to do, the challenge comes when the medical doctor disagrees with the midwife when the doctor is the one who is supposed to act and doesn't want to listen to the midwife. We however still contact the second on call, the specialist but still it delays intervention*”. FGD1 said

### Contributory factors to maternal deaths

The HCWs described that there are patient and facility related factors contributing to maternal deaths. The patient factors included non-attendance of antenatal services, non-use of contraceptives, unplanned pregnancies, late registration for antenatal care, and delay in seeking medical care, self-induced illegal abortions, and septic abortions. The views were espoused in the following statements:

“*There are very few patient factors, patients attend antenatal care services and they are well informed about their conditions. However, women need to plan for pregnancies in this way there won't be any self-induced abortions which ends up with sepsis*”. FGD1 “*Patients present themselves very late to health facilities, some register for antenatal care very late. Some women fall pregnant when they were not ready to fall pregnant. Late referrals and late identification of problems by referring facilities; delays in making decisions, One died of PIH, we had not done serial blood tests and induction of labour was not based on investigations*” said HCWD003.

HCWD002: “*Non registration for antenatal care, non-use of contraceptives Noncompliance to long-term medications (ARVs), Health care workers' substandard care; not following protocols, delays in instituting interventions, misdiagnosis patients*”.

The facility related factors identified include delayed interventions, mismanagement of patients at lower facilities, delays in referring patients, misdiagnosis, lack of skills, and non-consultation with others when dealing with complicated cases and failure to follow EMOC protocols. These were expressed in the quotes below:

HCW 001said: “*Poor management at lower facilities, you find a woman who attended antenatal care regularly are referred with complicated conditions which could have been identified and treated on time*”.

HCW024 said: “*The main problem currently is failure to follow EMOC protocol by some doctors and midwives, we see these things when we audit maternal deaths. Basically it is mismanagement, women die in our hands. We used to say it is shortage of staff but they die while we are attending to them because we did not do much*”.

### What could be done to reduce maternal deaths by different stakeholders?

The participants were of the opinion that majority of women attend maternal and child health care services and that men should play an active role in matters of child bearing and child rearing; and should be well informed and should attend such services with their partners. Similarly, communities should be supportive to pregnant women:

HCW013 said: “*I think women and their partners are doing well. Women register early for antenatal care and attend regularly. We fail them. I remember one woman visited the local clinic several times complaining of headache and each time she was given paracetamol; she then went for spiritual services and she had an eclamptic fit, upon arrival to the hospital, she died, this is very sad and painful*”.

HCW009 said: “*As health care workers we are to go back to working with key people in communities on health matters so that they can also impart appropriate information to women and their partners*”

HCWD001 said “*Nowadays it is rare to see nurses or midwives giving health talks in health facilities. These used to be done routinely and were helpful in empowering women about different conditions particularly in relation to pregnancy labour and delivery. We need to go back to old ways of doing things which benefited the community and the health care system*”

“*The community has to be supportive to women on sexual reproductive matters; communities have to avoid stigmatising pregnant women and accept them in their different conditions or situations. For example if a teenager falls pregnant, she needs to be supported so that she will not think of self-induced abortion. Communities need to encourage their members to use health care services available*” said HCW017.

HCWM002 said: “*We need to improve on our preparedness for emergencies, reduce unnecessary delays, and strengthen health education to women and their partners from the lowest facility to the referral hospital*”.

Our findings also indicate the need for policy makers to prioritise EMOC in-service training so that all relevant health care workers are trained; ensure availability of resources, address shortage of staff and allocate resources where they are critically needed, increase duration of training and review of policies.

“*The Ministry needs to give prevention of maternal deaths the attention it deserves, prioritize EMOC training as it is done with Safe male circumcision and PMTCT. For example, there used to be a Chief EMOC facilitator and currently there is none. Because of this, this year there were only 2 trainings and we used to have many which lasted for 2 weeks*”. FGD 2

“*They need to review old policies in line with current practices and availability of health facilities across the country. For example that prim gravidae should deliver in referral hospitals; that was when primary and district hospitals were not there. This needs to be changed because referral hospitals are always full of cases that could have been attended in lower facilities, and referral hospitals are always full because of such policies*”. FGD 3

HCWD004: *Women need to be empowered. Abortion needs to be legalised. Women present late with abortions, either already in sceptic shock or with sceptic abortion. They need to use contraceptives and they are offered for free. Use of contraceptives should be promoted even more for example there could be a national contraceptive day*.

## Discussion

The correlation between MMR and training has resulted in the recommendation that training of HCWs on EMOC is important for improving the standard of care and reducing MMR^13^,^14^,^16^. The authors indicated that simulation in teaching followed by practice were superior to traditional clinical and didactic education, and promotes skills acquisition and maintenance. Intensification of EMOC training and structuring maternal audits in Botswana aimed to reduce MMR. We found that only a handful of staff at PMH and NRH were trained on EMOC and participants expressed concern on the two week duration of training as being inadequate and suggested be increased so that healthcare workers could have more time to practice and also have an opportunity to attend to patients under supervision of the facilitators/experts. While concerns were expressed on the training duration, participants indicated that the training had actually improved the staff level of responsiveness to emergencies; confidence in managing haemorrhages and eclampsia. These findings are in contrast to reported findings that improvement in knowledge or skills after training does not translate to improved service delivery^17^ but supported by others who reported that emergency obstetric care in-service training is associated with improved knowledge and skills for healthcare providers working in maternity units^18^,^19^.

Participants in this study listed several factors they considered affecting EMOC implementation and categorised them into patient and facility related factors. The major patient related factors cited were non-attendance of antenatal services, non-use of contraceptives, unplanned pregnancies, late registration for antenatal care, and delay in seeking medical care, self-induced illegal abortions, septic abortions and underlying medical conditions. These findings are consistent with previous report in Zambia^20^. Antenatal Care services (ANC) were introduced as strategy to prevent and identify pregnancy risks in order to institute timely and appropriate management of pregnancy risks and treatment of the conditions^21^,^22^. The cited patient factors in this study are not consistent with the ANC objectives but suggest inadequacy in the community awareness on the need for pregnant mothers to access ANC services early and regularly so that risks can be identified and timely attended to. The self-induced illegal abortion and septic abortions continue to feature among the causes of maternal deaths in Botswana. These deaths may be related to restrictive legislations as abortion is illegal except under certain conditions^23^,^24^. Improving awareness using different approaches, multi-stakeholder involvement including community leaders, teachers and health professionals and review of legislation on abortion are likely to yield positive results.

The facility related factors cited included shortage of staff, delays in referring patients by lower facilities, non-attendance of antenatal care services, refusal to follow EMOC guidelines by some health care workers, delays in instituting interventions, unavailability of some drugs and supplies, negative attitude of some health care workers. Similar factors were reported previously^25^,^19^, who concluded that staff shortage, resistance to change, doctor-nurse hierarchy have negative effects on the quality of obstetric care. These factors are avoidable and require commitment and robust intervention strategies because healthcare workers are expected to provide quality of care that minimizes the risk of adverse maternal and new-born outcomes by providing prompt evidence based actions at the point of contact with the pregnant or recently delivered woman^18^,^26^. They however require resources that will empower them to deliver quality services. The government and health facility management should be made aware of these challenges and intensify EMOC training to more of the staff working in obstetrics and gynaecology departments in all facilities; and regularise in-services courses in order to sustain the skills and practices acquired during training.

The participants further expressed concern on involvement of partners and communities in reproductive health issues. They stated that while pregnant women attend ANC services, the male participation is minimal. In addition, they indicated the need for healthcare providers to work closely with families and communities in promoting maternal and child health; change attitudes towards work and in developing intervention strategies. Archibong & Agan^27^ have emphasized this need to strengthen community involvement in promoting maternal and child health and preventing morbidity and mortality. In support of community involvement, Gibore, Bali & Kibusi^28^ have indicated that male involvement is critical in preventing maternal deaths, and Pattinson et al. 29 share similar views that strengthening health systems, emergency obstetric care, establishing community linkages and increasing quality and equity of healthcare are the most effective in addressing maternal mortality. We suggest that couples should plan for pregnancies, male partners should be supportive to their partners, and women should take charge in reproductive decisions. For women with chronic conditions, they should consult health care workers for guidance when they plan to become pregnant.

Policy makers play a critical role in determining the effectiveness of the health care system as they plan and make final decisions on what is needed by the health care system. Okonofua et al.^30^ emphasises the need for policy makers to prioritise implementation of emergency obstetric care and deploy resources where they are most needed; and concentration on review and development of institutional policies, staff training, and uptake of standard practice guidelines and increased funding of maternal health services. Jamila, Hinda, & Östergren^25^ also underscores the need for policies and protocols for maternal deaths reviews, and the importance of maternal audits in identifying limitations and areas of improvement has been reported^31^,^32^. The views of the participants in this study indicate low priority is given to the prevention of maternal deaths compared to Safe Male Circumcision and PMTCT and on staff training on EMOC. In the view of the high MMR in Botswana, key stakeholders (government, hospital management) should give priority to EMOC in-service training so that all practitioners in the obstetrics and gynaecology departments are trained; assess adequacy of the training content, mode of delivery and duration of training, allocate adequate resources for supplies, address the shortage of staff and regularise maternal mortality audits in the health facilities.

## Conclusion and recommendations

EMOC in-service training improved knowledge and skills of health care workers resulting in improved service delivery and the need to strengthen health education in facilities cannot be overemphasized. Multi-stakeholder involvement is needed and policy makers should prioritize EMOC in-service training and its implementation and regular monitoring and evaluation are likely to result in a reduction in the national maternal mortality.
